# Effectiveness of Technology-Based Interventions for School-Age Children With Attention-Deficit/Hyperactivity Disorder: Systematic Review and Meta-Analysis of Randomized Controlled Trials

**DOI:** 10.2196/51459

**Published:** 2023-11-21

**Authors:** Ka Po Wong, Jing Qin, Yao Jie Xie, Bohan Zhang

**Affiliations:** 1 Department of Applied Social Sciences The Hong Kong Polytechnic University Hong Kong China (Hong Kong); 2 School of Nursing The Hong Kong Polytechnic University Hong Kong China (Hong Kong)

**Keywords:** attention-deficit/hyperactivity disorder, school-age children, computer-assisted training program, ADHD, neurofeedback training, virtual reality, cognitive functions

## Abstract

**Background:**

Attention-deficit/hyperactivity disorder (ADHD) is relatively common among school-age children. Technology-based interventions, such as computer-assisted training programs, neurofeedback training, and virtual reality, show promise in regulating the behaviors and cognitive functions of children with ADHD. An increasing number of randomized controlled trials have been conducted to evaluate the effectiveness of these technologies in improving the conditions of children with ADHD.

**Objective:**

This study aims to conduct a systematic review of technological interventions for school-age children with ADHD and perform a meta-analysis of the outcomes of technology-based interventions.

**Methods:**

A total of 19 randomized controlled studies involving 1843 participants were selected from a pool of 2404 articles across 7 electronic databases spanning from their inception to April 2022. ADHD behaviors, cognitive functions, learning ability, and quality of life were addressed in this study.

**Results:**

Random effects meta-analyses found that children with ADHD receiving technology-based intervention showed small and significant effect sizes in computer-rated inattention (standardized mean difference [SMD] −0.35; *P*<.04), parent-rated overall executive function measured by the Behavior Rating Inventory of Executive Function (SMD −0.35; *P*<.04), parent-rated disruptive behavior disorder measured by the Child Behavior Checklist (SMD −0.50; *P*<.001) and Disruptive Behavior Disorder Rating Scale (SMD −0.31; *P*<.02), and computer-rated visual attention measured by the Continuous Performance Test (SMD −0.42; *P*<.001) and Reaction Time (SMD −0.43; *P*<.02).

**Conclusions:**

Technology-based interventions are promising treatments for improving certain ADHD behaviors and cognitive functions among school-age children with ADHD.

**Trial Registration:**

PROSPERO CRD42023446924; https://tinyurl.com/7ee5t24n

## Introduction

### Background

Attention-deficit/hyperactivity disorder (ADHD) is one of the most common neurodevelopmental disorders among school-age children [1], with a prevalence of 7.6% [2]. Meanwhile, according to Diagnostic and Statistical Manual of Mental Disorders Text Revision Fourth Edition [3], the prevalence of ADHD in school-age children is 3% to 7% [3]. The 3 main categories of ADHD symptoms are inattention, hyperactivity, and impulsivity, which usually manifest in the school-age period [4]. These symptoms have detrimental impacts on the quality of life and functioning, including self-esteem, academic performance, social functioning, and relationship building [5]. ADHD is usually associated with long-term disability [6]. The types of treatments vary in different stages of life [7]. Behavioral parenting training and medication are the common approaches used to improve the behaviors and self-control of school-age children with ADHD [7]. ADHD medications are associated with an increased risk of headache, anxiety, and sleep disturbances [8,9]. Behavioral therapies are generally limited by time and space [10]. Therefore, feasible nonpharmacological approaches are recommended as alternatives to regulate the behaviors, executive functions, and well-being of children with ADHD.

In the last 10 years, human-computer interaction has widely been recognized in psychiatric and mental health research [11,12]. Digital technologies, neurofeedback systems, and virtual reality for health support, care, and treatment have increasingly been adopted, thus successfully gaining psychological health advantage [13,14]. ADHD is one of the common psychiatric disorders for which technology-based treatments are often used as therapeutic tools [15,16]. The application and effectiveness of technology-based interventions have been evaluated in ADHD treatment [4,17,18]. The advantages of technology-based treatment include improved executive functions and increased physiological and mental well-being [19,20]. However, inconsistent results have been reported regarding the efficiency of technology-based intervention in school-age children with ADHD. Regarding ADHD behaviors, Dovis et al [19], Egeland et al [21], Steiner et al [22], and van der Oord et al [23] found that computer-based training improved the inattention and hyperactivity in children with ADHD. However, some studies did not find significant results between technological treatment and ADHD behaviors [24-26]. Regarding executive functions, technology-based treatments improved inhibition [27], working memory [19,28], flexibility [19,27], emotional control [19], initiation [19], planning and organization [19], organizing materials [19], monitoring [19], and metacognition [22,23]. However, several studies determined that technology-based interventions have no effect on executive functions [21,24,25]. Regarding disruptive behavior disorder, Dovis et al [19], Lim et al [29], Steiner et al [22], and van der Oord et al [23] reported that computer-assisted training and neurofeedback training regulated oppositional defiant disorder and conduct disorder, whereas Bikic et al [24] and Breider et al [30] found no significant effect. Some studies have discovered that technology-based interventions can significantly improve visual attention [20,25,31], yet numerous studies indicated no significant effects [21,24,28,32,33]. These contradictory results make it difficult to examine the effectiveness of technology-based interventions in school-age children with ADHD.

Previous reviews illustrated the effectiveness of different types of technologies on children with ADHD [34-36]. Cibrian et al [34] and Powell et al [35] summarized this topic through a narrative description. Although Powell et al [35] adopted a meta-analysis to synthesize previous studies, their research focused on the use of virtual reality among children and adolescents with ADHD.

### Objective

These existing studies based on nonrandomized, cross-sectional, and observational designs have added to the knowledge base and identified the potential implications of technology in enhancing the ability and functions of children. In addition, more randomized controlled trials (RCTs) have been conducted in the recent decade, as a growing number of researchers have shown interest in the use of technology-based interventions to improve the capability and well-being of children with ADHD. A meta-analysis of RCTs can provide strong and robust evidence regarding the effectiveness of technology-based interventions in improving children with ADHD. Therefore, a systematic review and meta-analysis of the existing evidence of RCTs are needed to explicate the advantages of technology to school-age children with ADHD.

## Methods

### Search Strategy

This review focused on RCTs using technologies to regulate the ability and alleviate the well-being of children with ADHD. This review was registered in the PROSPERO International Prospective Register of Systematic Reviews (registration number CRD42023446924). The studies included in this review were searched from electronic databases, including PubMed, MEDLINE, ScienceDirect, Web of Science, CINAHL (via EBSCO), PsycINFO (via OVID), and Scopus, in April 2022. The keywords used in the search engines were as follows:

- Population: “children with ADHD” or “school-age children with ADHD” or “students with ADHD”
- Intervention: “technology” or “computer” or “robots” or “virtual reality” or “VR” or “augmented reality” or “AR” or “web-based” or “serious games”
- Outcomes: “inattentive” or “hyperactive-impulsive” or “hyperactivity” or “impulsivity” or “executive functions” or “executive functioning” or “inhibition” or “working memory” or “emotional control” or “flexibility” or “attention” or “initiation” or “planning” or “organization” or “time management” or “metacognition” or “quality of life” or “performance”

Multimedia Appendix 1 shows the full search strategy.

### Eligibility Criteria

The titles and abstracts of the selected papers were screened to identify relevant papers for this review. The analysis was performed using Population, Intervention, Control, and Outcomes framework (1) population: patients who were diagnosed with ADHD and aged between 6 and 12 years; (2) intervention: using technology (ie, the method of applying scientific knowledge for practical purposes) without restriction of technology type or frequency of intervention; (3) comparison: technology-based interventions for managing ADHD versus no interventions, interventions with placebo effect, and treatment as usual or waitlist control; and (4) outcome: behaviors, cognitive functions, and whole well-being evaluation results for patients with ADHD. Studies were selected using the following inclusion criteria: (1) articles published in English and (2) RCTs. Exclusion criteria included (1) teenagers, adults, and older adults; (2) comorbidity with autism spectrum disorder, psychosis, and affective or anxiety disorder; (3) consumption of toxic substances; (4) diagnosed with learning disorder; (5) non–peer reviewed studies; and (6) qualitative studies, reviews, cross-sectional studies, case studies, observational studies, study protocols, pre-post studies without a control group, or conference abstracts without full text. Furthermore, the references of the included papers were manually checked for eligibility. After removing duplicate articles, the studies were independently screened by 2 reviewers (KPW and BZ). The selected full-text articles were retrieved and reviewed by 4 reviewers (KPW, JQ, YJX, and BZ).

### Quality Assessment

The risk of bias in each study were independently evaluated by 3 reviewers (KPW, JQ, and BZ) using the Cochrane Collaboration tool for assessing the risk of bias [37]. The PRISMA (Preferred Reporting Items for Systematic Reviews and Meta-Analyses) criteria were adopted for conducting this review [38]. Multimedia Appendix 2 demonstrates the PRISMA checklist. The criteria of the tool included (1) random sequence generation, (2) allocation concealment, (3) blinding of participants and personnel, (4) blinding of outcome assessment, (5) incomplete outcome data, (6) selective reporting, and (7) other bias. In addition, judgment has 3 levels, including “low risk of bias,” “high risk of bias,” and “unclear risk of bias.” The conflicting results were settled by 4 reviewers (KPW, JQ, YJX, and BZ) through discussion.

### Data Extraction

The information of the selected studies was extracted and coded into different categories, including study characteristics (first author’s name, publication year, country, and setting), characteristics of participants (sample size, sex, and age), intervention and control condition (type of technology used, frequency, length, and duration), outcome measurement (rating scale, test, and questionnaire), and result (mean and SD).

### Data Synthesis

Data processing and analysis were conducted using the Review Manager Software RevMan (version 5.4), Cochrane Collaboration. The standardized mean difference (SMD) with a 95% CI was used to compute the effect size of the continuous outcomes of the interventions. The mean value of the baseline and posttest with SDs and the number of participants in the intervention groups and control groups were selected for the effect size calculation (ie, effect size of group differences). If reported, we selected the results estimated by the analysis of covariance, which treats individual baseline scores as covariates to correct for regression to baseline imbalanced means [39]. If the analysis of covariance had not been reported, change from baseline with SDs and posttest with SDs were selected. Multiple effect sizes were included in the same study, which contradicted the assumption that the effect sizes are independent of each other in the conventional meta-analytic procedures because the effect sizes in one study may be more correlated than those in other studies [40]. The results would become biased if this dependency was not considered. Hence, effect sizes assessed by the same measures were clustered to estimate the association between technology-based interventions and the conditions of school-age children with ADHD. The random effects model was applied in the meta-analysis, given the methodological diversity across the studies. Heterogeneity was measured using the *I*^2^ value (*P*<.10; *I*^2^>50%), and the higher the value of *I*^2^, the higher the level of heterogeneity. Subgroup analysis of parent-rated, teacher-rated, computer-rated, and self-rated results was performed, where applicable. To test moderating effects, 5 study-level characteristics, including the number of sessions, sample size, setting, game elements, and types of control group, were selected to calculate the meta-regression for each moderator.

## Results

### Selected Articles

**Figure 1:**
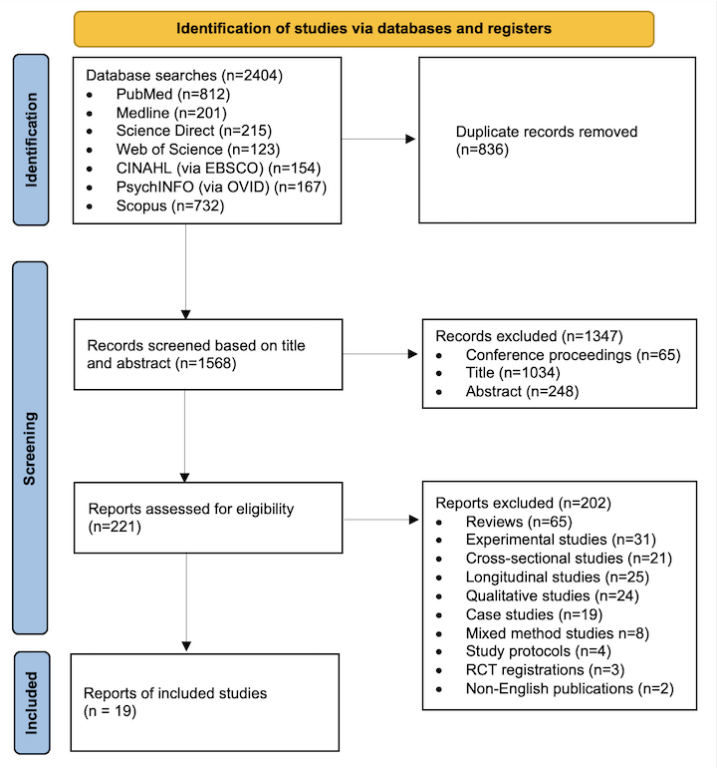
PRISMA (Preferred Reporting Items for Systematic Reviews and Meta-Analyses) flowchart of the study selection process. RCT: randomized controlled trial.

A total of 2404 articles were retrieved from electronic databases. After removing duplicates, the titles and abstracts of 1568 articles were screened. In total, 1347 articles were excluded, and the remaining 221 articles were selected for full-text screening. At this stage, we excluded reviews (n=65), experimental studies (n=31), cross-sectional studies (n=21), longitudinal studies (n=25), qualitative studies (n=24), case studies (n=19), mixed methods studies (n=8), study protocols (n=4), RCT registrations (n=3), and non-English publications (n=2). Finally, 19 RCTs that met the inclusion and exclusion criteria were identified. A flowchart of the study selection process is shown in Figure 1.

### Risk of Bias

**Figure 2:**
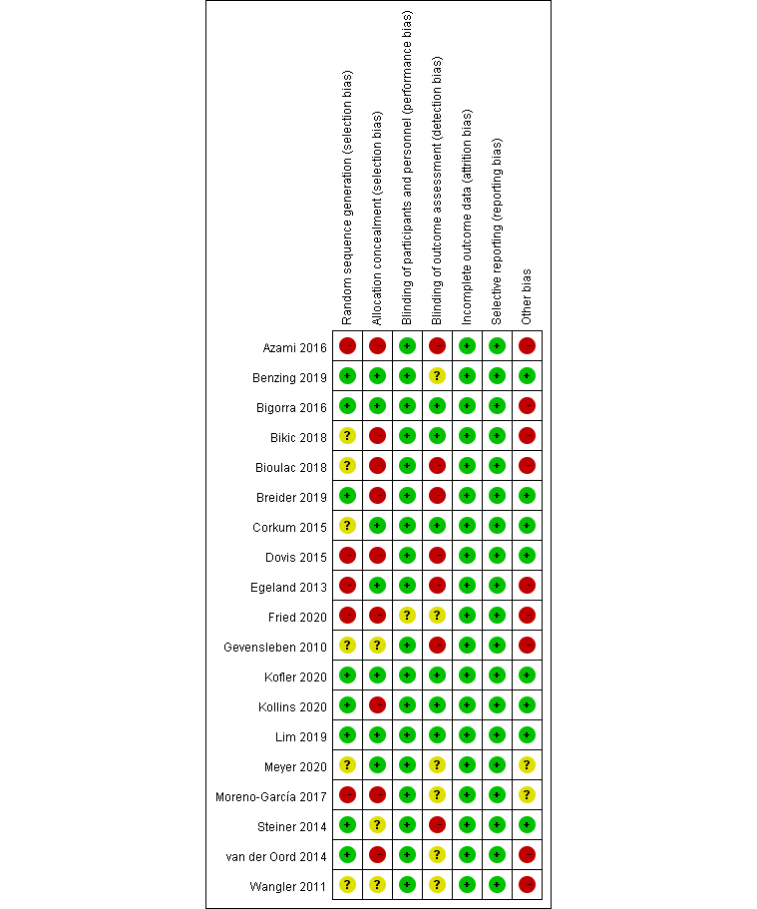
Risk of bias summary.

**Figure 3:**
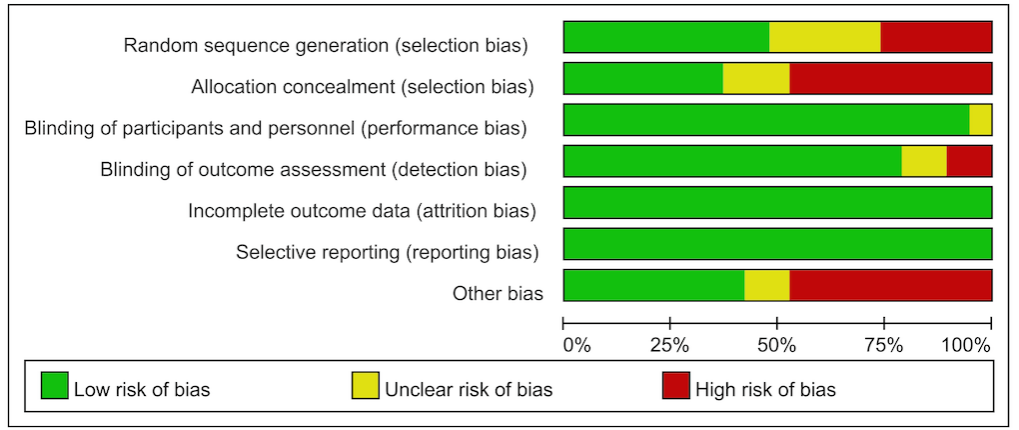
Risk of bias graph.

A low risk of random sequence generation was recorded from 8 trials (ie, the use of a computer random number generator and web-based system). Seven studies reported a low risk of allocation concealment. Meanwhile, 18 studies had a low risk of blinding participants and personnel. Although some studies had no complete blinding of participants and personnel [20-22,24,30,33,41,42], the reviewers determined that the outcomes were not likely to be affected by a lack of blinding. Six studies were judged to have a low risk of blinding the outcome assessment. Seven studies indicated no blinding of assessors, and 6 studies did not clearly indicate the blinding of assessors. All studies were judged to have a low risk of incomplete outcome data because the attrition rate of all studies was <20%. All studies were judged to have a low risk of selective reporting, as nearly all studies had the protocol and all studies reported the primary and secondary outcomes. Ten studies described the way to manage missing data (ie, intention-to-treat analysis) [19,22-25,27,29,30,41,43]. The risk of bias assessment is shown in Figures 2 and 3 [19,22-25, 27,29,30,41,43].

### Study Characteristics

Fourteen studies used a 2-arm trials design [19-27,29-32,41-44], and the remaining 5 studies had a 3-arm trial design [19,20,22,28,33]. These studies were conducted in 11 countries: Canada [41], Denmark [24], France [33], Germany [31,42], Iran [28], the Netherlands [19,23,30], Norway [21], Singapore [29], Spain [20,32], Switzerland [27], and the United States [22,25,26,43,44].

### Participants Characteristics

A total of 1843 school-age children were included in this review, all of whom were diagnosed with ADHD. The number of participants included in each trial ranged from 10 to 246. Most studies were conducted in participants’ homes, and a small number of studies were conducted in schools, classrooms, and clinics. Detailed information is presented in Table 1.

**Table 1:** Participants’ characteristics of the selected studies (n=19).

Study	Country	Setting	Sample size, n	Sex (female/male), n/n	Age (years), mean (range)^a^
Azami et al [28]	Iran	Participants’ home	34	0/34	7-12
Benzing and Schmidt [27]	Switzerland	Participants’ home	51	10/41	10.43 (8-12)
Bigorra et al [32]	Spain	Participants’ home	66	36/24	8.9
Bikic et al [24]	Denmark	Participants’ home	70	11/59	9.96
Bioulac et al [33]	France	No mention	51	10/41	8.9
Breider et al [30]	Netherlands	Participants’ home	21	6/15	7.76
Corkum et al [41]	Canada	Classroom-based	58	7/51	8.83 (6-12)
Dovis et al [19]	Netherlands	Home-based training	89	18/71	10.50
Egeland et al [21]	Norway	School	67	18/49	10.4
Fried et al [44]	The United States	Participants’ home	333	88/245	9.13
Gevensleben et al [42]	Germany	No mention	94	17/77	9.21
Kofler et al [25]	The United States	Office and participants’ home	54	12/42	10.41
Kollins et al [43]	The United States	Participants’ home	348	100/248	9.65
Lim et al [29]	Singapore	Clinic	172	25/147	8.6
Meyer et al [26]	The United States	Home	40	12/28	10.15
Moreno-García et al [20]	Spain	Home and school	57	13/44	8.84
Steiner et al [22]	The United States	Classroom	104	34/70	8.56
van der Oord et al [23]	Netherlands	Home	40	7/33	9.75
Wangler et al [31]	Germany	No mention	94	17/77	9.64

^a^If studies did not provide mean values and/or ranges for participant ages, this information was not shown in the table.

### Technology-Based Intervention and Control Condition

Different types of technologies were adopted in the intervention group among the included studies, including computer-assisted training programs, neurofeedback training, and virtual reality. For the control groups, 6 studies used treatment or medication as the usual approach. The participants in the control groups of 3 included studies did not receive any training. Two studies used stimulants, 7 studies used placebo cognitive training, and 1 study used a digital game. Detailed information is presented in Table 2.

**Table 2:** Characteristics of intervention of the selected studies (n=19).

Study	Intervention	Control	Type of intervention	Outcome measurement
Azami et al [28]	Computer-assisted cognitive rehabilitation	Psychostimulants	Individual: 20 sessions in 3 mo, 90 min/session	Continuous Performance Test, Tower-of-London, Forward/Backward Digit Span From WISC-R^a^, Raven Progressive Matrices, and web-based version of Span Board Task Progressive Matrices
Benzing and Schmidt [27]	Exergame training	Not receiving training	Individual: 8 wk, 3 times a week for at least 30 min	Conners‐3 Rating Scales and German Motor Test
Bigorra et al [32]	Computerized working memory training	Nonadaptive work memory training	Individual: 5 wk, 5 sessions per week, 30-45 min/session	Backward Digit Span, Letter-number Sequencing of WISC-IV^b^, Backward Spatial Span of WMS-III^c^, Lowa Gambling Task, Happé Strange Stories, and Folk Psychology Test
Bikic et al [24]	Cognitive computer games of the ACTIVATE program	Treatment as usual	Individual: 6 times a week, 8 wk	Cambridge Neuropsychological Test Automated Battery, Motor Screening Task, Attention Switching Task, Rapid Visual Information Processing, Intraextra Dimensional Set Shift, Reaction Time, ADHD^d^ Rating Scale, and Behavior Rating Inventory of Executive Function
Bioulac et al [33]	Virtual classroom cognitive remediation program	Psychotherapy placebo training	Individual: twice a week for 6 wk, 12 sessions, 30 min/session	ADHD Rating Scale and Continuous Performance Test Task Assessment
Breider et al [30]	Web-based program and supportive therapist contact	Face-to-face parent training	Parents: 17 sessions, 45-60 min/session	Child Behavior Checklist
Corkum et al [41]	Web-based learning and blackboard learning	Treatment as usual	Teachers: 6 training sessions	Conners-3 Parent and Teacher Rating Scales and Impairment Rating Scale
Dovis et al [19]	Braingame Brian training	Placebo-mode working memory training	Individual: 25 training sessions in 5 wk, 35-50 min per session	Disruptive Behavior Disorder Rating Scale, Behavior Rating Inventory of Executive Function, Sensitivity to Punishment and Sensitivity to Reward Questionnaire for Children, Pediatric Quality of Life Inventory, and Home Situations Questionnaire
Egeland et al [21]	Cogmed RoboMemo program	Treatment as usual	Individual: daily basis for 5-7 wk, 30-45 min	Color Word Test, Trail Making Test, Conners Continuous Performance Test-II, Key Math, Logometrica, Benton Visual Retention Test, Children’s Auditory Verbal Learning Test-2, ADHD Rating Scale, Strengths and Difficulties Questionnaire, and Behavior Rating Inventory of Executive Function
Fried et al [44]	Text messaging	Treatment as usual	Individual: 45 d	Adherence to stimulants
Gevensleben et al [42]	Neurofeedback training	Attention skills training	Individual: 6 mo, 25-30 min/session	ADHD Rating Scale, German Rating Scale for Oppositional Defiant/Conduct Disorders, Strength and Difficulties Questionnaire, Home Situations Questionnaire, and Homework Problem Checklist
Kofler et al [25]	Central executive training	Inhibitory control training	Group: 10 wk, 1 h/wk; individual: 10 wk, 2-3 d/wk, 15 min/d	Behavior Assessment System for Children, ADHD Rating Scale, and Phonological and Visuospatial Reordering
Kollins et al [43]	AKL-T01 (a digital therapeutic)	Digital game	Individual: 5 d/wk for 4 wk, 25 min/d	ADHD Rating Scale, Test of Variables of Attention, and Attention Performance Index
Lim et al [29]	Brain computer interface-based attention training program	Not receiving training	Individual: 3 sessions/wk in the first 8 wk and 4 sessions/wk in the next 12 wk	ADHD Rating Scale Inattention Score and Child Behavior Checklist
Meyer et al [26]	Computerized training	Medication as usual	Individual: at least 5 d a week for 4 wk, 15 min/d	Swanson, Nolan, and Pelham-IV Questionnaire and Conners Parent Rating Scale and Conners Teacher Rating Scale
Moreno-García et al [20]	Neurofeedback training	Pharmacology	Individual: 40 sessions	ADHD Rating Scale and Integrated Visual and Auditory Continuous Performance Test
Steiner et al [22]	Computer attention training using neurofeedback or cognitive training	Not receiving training	Individual: 40 sessions over 5 mo, 3 times/wk, 45 min/time	Conners 3–Parent Assessment Report, Behavior Rating Inventory of Executive Function, and Behavioral Observation of Students in Schools
van der Oord et al [23]	Executive functioning training	Treatment as usual	Individual: 25 sessions over 5 wk, 40 min/session	Behavior Rating Inventory of Executive Functioning and Disruptive Behavior Disorder Rating Scale
Wangler et al [31]	Neurofeedback training	Computerized attention skills training	Individual: 3-4 wk, 25-30 min/session	Attention Network Test and ADHD Rating Scale

^a^WISC-R: Wechsler Intelligence Scale for Children—Revised.

^b^WISC-IV: Backward Digit Span of the Wechsler Intelligence Scale for Children-IV.

^c^WMS-III: Wechsler Memory Scale–III.

^d^ADHD: attention-deficit/hyperactivity disorder.

### Intervention Duration and Length

The intervention duration of 3 studies was 4 weeks. Meanwhile, the intervention duration of 9 studies was 5 to 8 weeks. One study had an intervention duration of 12 weeks, 2 studies had an intervention duration of 5 months, and 1 study had an intervention duration of 6 months. For the length of intervention, 1 trial was conducted for 15 minutes per session, 10 studies varied from 25 to 45 minutes per session, 2 studies varied from 45 to 60 minutes per session, and 1 study was conducted for 90 minutes per session.

### Meta-Analysis Results of the Technology-Based Intervention

#### ADHD Behaviors

ADHD behaviors included in this study were inattention and hyperactivity or impulsivity. Moderate heterogeneity was observed among the studies examining ADHD behaviors (*I*^2^=26.4%). Table 3 shows the pooled results of ADHD behavior. Corkum et al [41] evaluated the overall ADHD behaviors of participants, whereas the other 10 studies classified the results of the ADHD behaviors into inattention and hyperactivity or impulsivity. Corkum et al [41] reported the Conners 3 Parent Rating Scale (Conners 3-P) and Conners 3 Teacher Rating Scale (Conners 3-T) scores. The SMD of ADHD behaviors measured with parent-rated evaluation was −0.21 (95% CI −0.73 to 0.31), and the teacher-rated evaluation of ADHD behaviors was −0.46 (95% CI −0.98 to 0.06). However, no significant effect was observed for ADHD behavior.

A total of 12 studies reported inattention, which was measured using the ADHD Rating Scale (ADHD-RS), Disruptive Behavior Disorder Rating Scale (DBDRS), Conners 3-P, and Conners 3-T. Parent-rated inattention measured by ADHD-RS (*K*=7), Conners 3-P (*K*=4), and DBDRS (*K*=2) had no statistically significant effect. Teacher-rated inattention measured by ADHD-RS (*K*=6), Conners 3-T (*K*=1), and DBDRS (*K*=2) had no significant effect. Computer-rated inattention measured by ADHD-RS (*K*=2; SMD −0.35, 95% CI −0.68 to −0.01) had a small and statistically significant effect (*P*<.04).

Ten studies explored the effectiveness of technology-based interventions on hyperactivity or impulsivity. Three different scales were used: ADHD-RS, DBDRS, and Conners 3-P and Conners 3-T. No significant effect was found in parent-rated hyperactivity or impulsivity measured by ADHD-RS (*K*=7), Conners 3-P (*K*=4), and DBDRS (*K*=2) and teacher-rated hyperactivity or impulsivity measured by ADHD-RS (*K*=4), Conners 3-T (*K*=1), and DBDRS (*K*=2).

The results of moderator analysis demonstrated that the sample size, setting, game elements, and type of control group moderated the effect size (Table 4). A sample size of ≤50 (*K*=10; SMD −0.25, 95% CI −0.47 to −0.03; *P*<.03), nonhome setting (ie, clinic or school; *K*=12; SMD −0.24, 95% CI −0.37 to −0.11; *P*<.001), game elements excluded (*K*=40; SMD −0.15, 95% CI −0.23 to −0.07; *P*<.001), and control groups with no treatment (*K*=28; SMD −0.22, 95% CI −0.32 to −0.13; *P*<.001) and nonequivalent treatment had a significant moderating effect on the effect size.

**Table 3:** Meta-analyses results of technology-based interventions for school-age children with attention-deficit/hyperactivity disorder.

Variables			Number of effect sizes		Standardized mean difference		SE		95% CI		*z* score		*P* value
**Parent-rated inattention**													
	ADHD-RS^a^	7		−0.190		0.099		−0.384 to 0.004		−1.92		.05	
	Conners 3-P^b^	4		−0.373		0.144		−0.655 to −0.092		−2.60		.10	
	DBDRS^c^	2		−0.462		0.212		−0.878 to −0.045		−2.17		.09	
**Teacher-rated inattention**													
	ADHD-RS	6		0.052		0.077		−0.098 to 0.202		0.678		.50	
	Conners 3-T^d^	1		−0.150		0.318		−0.774 to 0.474		−0.472		.64	
	DBDRS	2		−0.113		0.201		−0.507 to 0.280		−0.564		.57	
**Computer-rated inattention**													
	ADHD-RS	2		−0.345		0.17		−0.679 to −0.011		−2.025		.043	
**Parent-rated hyperactivity or impulsivity**													
	ADHD-RS	7		0.032		0.14		−0.243 to 0.306		0.225		.82	
	Conners 3-P	4		−0.16		0.184		−0.521 to 0.200		−0.871		.38	
	DBDRS	2		−0.311		0.267		−0.834 to 0.212		−1.164		.24	
**Teacher-rated hyperactivity or impulsivity**													
	ADHD-RS	4		0.097		0.133		−0.163 to 0.358		0.732		.46	
	Conners 3-T	1		0.312		0.318		−0.312 to 0.935		0.979		.33	
	DBDRS	2		−0.13		0.2		−0.521 to 0.261		−0.651		.52	
**Parent-rated executive functions**													
	BRIEF^e^	2		−0.347		0.17		−0.681 to −0.013		−2.038		.04	
**Parent-rated inhibition**													
	BRIEF	3		−0.192		0.192		−0.568 to 0.185		−0.998		.32	
	German Motor Test	1		−0.686		0.349		−1.370 to −0.002		−1.967		.05	
**Computer-rated inhibition**													
	BASC^f^	1		0.231		0.274		−0.306 to 0.768		0.844		.40	
	BRIEF	1		0.125		0.273		−0.410 to 0.661		0.458		.65	
**Parent-rated working memory**													
	BRIEF	3		−0.032		0.154		−0.333 to 0.269		−0.206		.84	
	German Motor Test	1		0.285		0.283		−0.269 to 0.840		1.009		.31	
	WISC-IV^g^	1		0.788		0.266		0.267 to 1.309		2.965		<.001	
**Computer-rated working memory**													
	BRIEF	2		0.261		0.449		−0.620 to 1.141		0.580		.56	
	WISC-IV	2		1.486		0.534		0.439 to 2.534		2.782		.01	
**Parent-rated flexibility**													
	BRIEF	3		−0.142		0.259		−0.649 to 0.365		−0.549		.58	
	German Motor Test	1		−0.566		0.457		−1.462 to 0.331		−1.237		.22	
**Parent-rated emotional control**													
	BRIEF	2		0.035		0.419		−0.786 to 0.856		0.084		.93	
**Parent-rated initiation**													
	BRIEF	2		0.108		0.244		−0.370 to 0.586		0.442		.66	
**Teacher-rated initiation**													
	BRIEF	2		0.196		0.169		−0.136 to 0.528		1.156		.25	
**Parent-rated planning and organization**													
	BRIEF	2		0.057		0.253		−0.438 to 0.553		0.227		.82	
**Parent-rated organizing materials**													
	BRIEF	2		0.118		0.177		−0.230 to 0.465		0.663		.51	
**Parent-rated monitoring**													
	BRIEF	2		0.291		0.327		−0.349 to 0.932		0.891		.37	
**Parent-rated metacognition**													
	BRIEF	5		−0.144		0.131		−0.400 to 0.113		−1.099		.27	
**Teacher-rated metacognition**													
	BRIEF	2		−0.116		0.171		−0.451 to 0.220		−0.676		.50	
**Parent-rated disruptive behavior disorder**													
	ADHD-RS	1		0.350		0.242		−0.124 to 0.825		1.447		.15	
	BOSS^h^	2		−0.187		0.170		−0.521 to 0.147		−1.096		.27	
	CBC^i^	3		−0.504		0.111		−0.720 to −0.287		−4.550		<.001	
	DBDRS	5		−0.306		0.125		−0.552 to −0.060		−2.441		.02	
	FBB-SSV^j^	3		−0.127		0.154		−0.428 to 0.174		−0.826		.41	
**Teacher-rated disruptive behavior disorder**													
	ADHD-RS	1		0.076		0.239		−0.392 to 0.545		0.319		.75	
	DBDRS	4		0.009		0.141		−0.267 to 0.286		0.065		.95	
**Computer-rated visual attention**													
	ANT^k^	5		0.014		0.112		−0.207 to 0.234		0.121		.90	
	AST^l^	2		−0.114		0.183		−0.473 to 0.244		−0.624		.53	
	BVRT^m^	1		0.000		0.263		−0.516 to 0.516		0.000		>.99	
	CPT^n^	8		−0.419		0.124		−0.662 to −0.176		−3.377		<.001	
	IED^o^	1		−0.313		0.260		−0.822 to 0.196		−1.205		.23	
	IVA/CPT^p^	6		−0.250		0.132		−0.508 to 0.009		−1.892		.06	
	RTI^q^	2		−0.425		0.186		−0.790 to −0.060		−2.280		.02	
	RVIP^r^	4		−0.021		0.130		−0.275 to 0.233		−0.165		.87	
**Computer-rated auditory attention**													
	CAVLT-2^s^	5		0.011		0.147		−0.276 to 0.298		0.074		.94	
	IVA/CPT	4		−0.084		0.195		−0.467 to 0.298		−0.431		.67	
**Parent-rated sensitivity to punishment and sensitivity to reward**													
	SPSRQ-C^t^	4		−0.147		0.128		−0.399 to 0.104		−1.148		.25	
**Parent-rated quality of life**													
	PedsQL^u^	1		0.646		0.263		0.131 to 1.161		2.458		.01	
**Self-rated quality of life**													
	PedsQL	1		0.042		0.256		−0.460 to 0.544		0.163		.87	
**Computer-rated reading fluency**													
	LOGOS^v^	2		0.014		0.186		−0.351 to 0.379		0.073		.94	
**Parent-rated adherence to stimulants**													
	Adherence to stimulants	1		−0.113		0.125		−0.358 to 0.132		−0.906		.37	

^a^ADHD-RS: ADHD Rating Scale.

^b^Conners 3-P: Conners 3 Parent Rating Scale.

^c^DBDRS: Disruptive Behavior Disorder Rating Scale.

^d^Conners 3-T: Conners 3 Teacher Rating Scale.

^e^BRIEF: Behavior Rating Inventory of Executive Function.

^f^BASC: Behavior Assessment System for Children.

^g^WISC-IV: Backward Digit Span of the Wechsler Intelligence Scale for Children-IV.

^h^BOSS: Behavioral Observation of Students in Schools.

^i^CBC: Child Behavior Checklist.

^j^FBB-SSV: Fremdbeurteilungsbogen für Störungen des Sozialverhaltens.

^k^ANT: Attention Network Test.

^l^AST: Attention Switching Task.

^m^BVRT: Benton Visual Retention Test.

^n^CPT: Continuous Performance Test.

^o^IED: Intraextra Dimensional Set Shift.

^p^IVA/CPT: Integrated Visual and Auditory Continuous Performance Test.

^q^RTI: Reaction Time.

^r^RVIP: Rapid Visual Information Processing.

^s^CAVLT-2: Children’s Auditory Verbal Learning Test-2.

^t^SPSRQ-C: Sensitivity to Punishment and Sensitivity to Reward Questionnaire for Children.

^u^PedsQL: Pediatric Quality of Life Inventory.

^v^LOGOS: Logometrica.

**Table 4:** Results of moderators between technology-based intervention for school-age children with attention-deficit/hyperactivity disorder (ADHD).

			Number of effect sizes	Standardized mean difference	SE	95% CI	*z* score	*P* value	Heterogeneity	
									Q-statistics	*I^2^*
**ADHD behavior**			46	−0.105	0.037	−0.177 to −0.033	−2.853	<.001	61.123	26.378
	**Number of sessions**		N/A^a^	N/A	N/A	N/A	−2.312	.02	N/A	N/A
		≤20	11	−0.012	0.094	−0.195 to 0.172	−0.124	.90		
		>20 to 40	26	−0.111	0.060	−0.229 to 0.007	−1.841	.07		
		>40 to 60	4	−0.163	0.143	−0.443 to 0.117	−1.140	.25		
		>60	2	−0.254	0.153	−0.554 to 0.045	−1.663	.10		
	**Sample size**		N/A	N/A	N/A	N/A	−2.651	.01	N/A	N/A
		≤50	10	−0.245	0.112	−0.465 to −0.025	−2.183	.03		
		>50 to 100	29	−0.076	0.057	−0.188 to 0.037	−1.320	.19		
		>100	7	−0.143	0.092	−0.324 to 0.037	−1.555	.12		
	**Setting**		N/A	N/A	N/A	N/A	−2.651	.01	N/A	N/A
		Home	21	−0.102	0.056	−0.212 to 0.007	−1.827	.07		
		Mixed	8	0.204	0.107	−0.006 to 0.414	1.905	.06		
		Nonhome	12	−0.242	0.066	−0.372 to −0.113	−3.670	<.001		
	**Game elements**		N/A	N/A	N/A	N/A	−2.800	.01	N/A	N/A
		Game elements excluded	40	−0.151	0.043	−0.234 to −0.068	−3.546	<.001		
		Game elements included	1	0.241	0.131	−0.015 to 0.497	1.845	.07		
	**Types of control group**		N/A	N/A	N/A	N/A	−2.756	.01	N/A	N/A
		Equivalent treatment	9	0.083	0.071	−0.057 to 0.223	1.161	.25		
		No treatment	28	−0.225	0.047	−0.317 to −0.132	−4.749	<.001		
		Nonequivalent treatment	4	0.337	0.165	0.013 to 0.661	2.041	.04		
**Executive functions**			48	0.013	0.037	−0.060 to 0.087	0.357	.72	99.361	52.698
	**Number of sessions**		N/A	N/A	N/A	N/A	0.257	.80	N/A	N/A
		≤20	3	0.777	0.303	0.183 to 1.371	2.564	.01		
		>20 to 40	43	−0.012	0.056	−0.121 to 0.097	−0.214	.83		
		>40 to 60	2	0.012	0.253	−0.484 to 0.508	0.048	.96		
	**Sample size**		N/A	N/A	N/A	N/A	0.260	.80	N/A	N/A
		≤50	7	−0.046	0.168	−0.376 to 0.284	−0.272	.79		
		>50 to 100	37	0.063	0.058	−0.051 to 0.177	1.082	.28		
		>100	4	−0.363	0.174	−0.704 to −0.023	−2.093	.04		
	**Setting**		N/A	N/A	N/A	N/A	0.257	.80	N/A	N/A
		Home	40	0.047	0.059	−0.068 to 0.163	0.802	.42		
		Mixed	2	0.178	0.268	−0.347 to 0.703	0.664	.51		
		Nonhome	6	−0.239	0.146	−0.525 to 0.047	−1.641	.10		
	**Game elements**		N/A	N/A	N/A	N/A	0.260	.80	N/A	N/A
		Game elements excluded	42	0.009	0.055	−0.099 to 0.116	0.161	.87		
		Game elements included	3	−0.318	0.219	−0.748 to 0.112	−1.448	.15		
	**Types of control group**		N/A	N/A	N/A	N/A	0.257	.80	N/A	N/A
		Equivalent treatment	11	−0.022	0.111	−0.240 to 0.196	−0.196	.84		
		No treatment	34	−0.007	0.062	−0.129 to 0.115	−0.118	.91		
		Nonequivalent treatment	3	0.777	0.303	0.183 to 1.370	2.565	.01		
**Disruptive behavior disorder**			19	−0.212	0.057	−0.323 to −0.101	−3.735	<.001	27.160	33.725
	**Number of sessions**		N/A	N/A	N/A	N/A	−3.307	<.001	N/A	N/A
		≤20	1	−1.070	0.475	−2.001 to −0.138	−2.251	.02		
		>20 to 40	13	−0.102	0.078	−0.254 to 0.050	−1.312	.19		
		>60	2	−0.470	0.128	−0.722 to −0.219	−3.668	<.001		
	**Sample size**		N/A	N/A	N/A	N/A	−3.422	<.001	N/A	N/A
		≤50	5	−0.395	0.156	−0.702 to −0.089	−2.527	.01		
		>50 to 100	10	−0.035	0.084	−0.200 to 0.131	−0.411	.68		
		>100	4	−0.377	0.102	−0.577 to −0.176	−3.675	<.001		
	**Setting**		N/A	N/A	N/A	N/A	−2.714	.01	N/A	N/A
		Home	12	−0.127	0.096	−0.314 to 0.061	−1.322	.19		
		Nonhome	4	−0.361	0.130	−0.017 to −0.616	−2.781	.01		
	**Game elements**		N/A	N/A	N/A	N/A	−2.738	.01	N/A	N/A
		Game elements excluded	19	−0.196	0.072	−0.337 to −0.056	−2.738	.01		
	**Types of control group**		N/A	N/A	N/A	N/A	−2.911	<.001	N/A	N/A
		Equivalent treatment	8	−0.106	0.106	−0.314 to 0.102	−1.001	.32		
		No treatment	10	−0.235	0.090	−0.412 to −0.058	−2.606	.01		
		Nonequivalent treatment	1	−1.070	0.491	−2.032 to −0.108	−2.179	.03		
**Visual attention**			29	−0.174	0.050	−0.273 to −0.076	−3.487	<.001	36.296	22.856
	**Number of sessions**		N/A	N/A	N/A	N/A	−3.415	<.001	N/A	N/A
		≤20	5	−0.355	0.185	−0.718 to 0.009	−1.914	.06		
		>20 to 40	18	−0.232	0.077	−0.383 to −0.080	−3.000	<.001		
		>40 to 60	1	0.000	0.307	−0.602 to 0.602	0.000	>.99		
	**Sample size**		N/A	N/A	N/A	N/A	−3.112	<.001	N/A	N/A
		≤50	2	−0.025	0.315	−0.642 to 0.592	−0.080	.94		
		>50 to 100	27	−0.187	0.059	−0.303 to −0.071	−3.152	<.001		
	**Setting**		N/A	N/A	N/A	N/A	−2.810	.01	N/A	N/A
		Home	12	−0.162	0.095	−0.349 to 0.025	−1.700	.10		
		Mixed	8	−0.323	0.127	−0.571 to −0.075	−2.550	.01		
		Nonhome	1	0.000	0.312	−0.612 to 0.612	0.000	>.99		
	**Game elements**		N/A	N/A	N/A	N/A	−3.146	<.001	N/A	N/A
		Game elements excluded	27	−0.187	0.059	−0.303 to −0.071	−3.152	<.001		
		Game elements included	2	−0.025	0.315	−0.642 to 0.592	−0.080	.94		
	**Types of control group**		N/A	N/A	N/A	N/A	−3.146	<.001	N/A	N/A
		Equivalent treatment	13	−0.267	0.086	−0.435 to −0.099	−3.109	<.001		
		No treatment	10	−0.147	0.089	−0.322 to 0.028	−1.647	.10		
		Nonequivalent treatment	6	−0.003	0.155	−0.306 to 0.300	−0.017	.99	—^b^	—
**Auditory attention**			9	−0.020	0.091	−0.199 to 0.159	−0.218	.83	11.641	31.279
	**Number of sessions**		N/A	N/A	N/A	N/A	−0.200	.84	N/A	N/A
		>20 to 40	4	−0.084	0.195	−0.467 to 0.298	−0.431	.67		
		>40 to 60	5	0.011	0.147	−0.276 to 0.298	0.074	.94		
	**Sample size**		N/A	N/A	N/A	N/A	−0.205	.84	N/A	N/A
		>50 to 100	9	−0.023	0.111	−0.240 to 0.195	−0.205	.84		
	**Setting**		N/A	N/A	N/A	N/A	−0.200	.84	N/A	N/A
		Mixed	4	−0.084	0.195	−0.467 to 0.298	−0.431	.67		
		Nonhome	5	0.011	0.147	−0.276 to 0.298	0.074	.94		
	**Game elements**		N/A	N/A	N/A	N/A	−0.205	.84	N/A	N/A
		Game elements excluded	9	−0.023	0.111	−0.240 to 0.195	−0.205	.84		
	**Types of control group**		N/A	N/A	N/A	N/A	−0.200	.84	N/A	N/A
		No treatment	5	0.011	0.147	−0.276 to 0.298	0.074	.94		
		Nonequivalent treatment	4	−0.084	0.195	−0.467 to 0.298	−0.431	.67		
Sensitivity to punishment and sensitivity to reward			4	−0.147	0.128	−0.399 to 0.104	−1.148	>.99	0.000	0.000
**Quality of life**			2	0.336	0.183	−0.023 to 0.696	1.833	.07	2.712	63.124
	**Number of sessions**		N/A	N/A	N/A	N/A	1.129	.26	N/A	N/A
		>20 to 40	2	0.341	0.302	−0.251 to 0.933	1.129	.26		
	**Sample size**		N/A	N/A	N/A	N/A	N/A	N/A	N/A	N/A
		>50 to 100	2	0.341	0.302	−0.251 to 0.933	1.129	.26		
	**Setting**		N/A	N/A	N/A	N/A	N/A	N/A	N/A	N/A
		Home	2	0.341	0.302	−0.251 to 0.933	1.129	.26		
	**Game elements**		N/A	N/A	N/A	N/A	N/A	N/A	N/A	N/A
		Game elements excluded	2	0.341	0.302	−0.251 to 0.933	1.129	.26		
	**Types of control group**		N/A	N/A	N/A	N/A	N/A	N/A	N/A	N/A
		Equivalent treatment	2	0.341	0.302	−0.251 to 0.933	1.129	.26		
**Reading fluency**			2	0.014	0.173	−0.326 to 0.353	0.079	.94	1.156	13.528
	**Number of sessions**		N/A	N/A	N/A	N/A	0.073	.94	N/A	N/A
		>40 to 60	2	0.014	0.186	−0.351 to 0.379	0.073	.94		
	**Sample size**		N/A	N/A	N/A	N/A	0.073	.94	N/A	N/A
		>50 to 100	2	0.014	0.186	−0.351 to 0.379	0.073	.94		
	**Setting**		N/A	N/A	N/A	N/A	0.073	.94	N/A	N/A
		Nonhome	2	0.014	0.186	−0.351 to 0.379	0.073	.94		
	**Game elements**		N/A	N/A	N/A	N/A	0.073	.94	N/A	N/A
		Game elements excluded	2	0.014	0.186	−0.351 to 0.379	0.073	.94		
	**Types of control group**		N/A	N/A	N/A	N/A	0.073	.94	N/A	N/A
		No treatment	2	0.014	0.186	−0.351 to 0.379	0.073	.94		

^a^N/A: not applicable.

^b^Not available.

#### Executive Functions

The fundamental skills of executive functions include inhibition, working memory, flexibility, emotional control, initiation, planning and organization, organizing materials, monitoring, and metacognition. Substantial heterogeneity was observed among the studies that examined executive functions (*I*^2^=52.7%). Table 3 shows the pooled results of the fundamental skills of executive functions. Steiner et al [22] reported an overall executive function of school-aged children with ADHD and SMD using the Behavior Rating Inventory of Executive Function (BRIEF) score. The SMD was −0.35 (95% CI −0.68 to −0.01), indicating a small and statistically significant effect was observed for the overall executive function.

Five studies reported inhibition through BRIEF, Behavior Assessment System for Children, and German Motor Test. Parent-rated inhibition using BRIEF (*K*=3) and German Motor Test (*K*=1) showed no significant effect. No significant effect was observed for computer-rated inhibition measured by BRIEF (*K*=1) and Behavior Assessment System for Children (*K*=1).

Six studies investigated working memory. Three different scales were used: the Backward Digit Span of the Wechsler Intelligence Scale for Children-IV (WISC-IV), BRIEF, and German Motor Test. No significant effect was observed for parent-rated working memory measured by BRIEF (*K*=3), German Motor Test (*K*=1), and WISC-IV (*K*=1). A large and statistically significant effect favoring the control group was observed for computer-rated working memory measured by WISC-IV (*K*=2; SMD 1.49, 95% CI 0.44-2.53).

Four studies evaluated flexibility using BRIEF and German Motor Test. No significant result was observed for parent-rated flexibility measured by BRIEF (*K*=3) and German Motor Test (*K*=1).

Two studies reported emotional control, and the BRIEF score was used for the measurement. No significant effect was observed for parent-rated emotional control and teacher-rated emotional control.

Initiation was reported by 2 studies using BRIEF to score. No statistically significant result was found for parent-rated initiation and teacher-rated initiation.

Three studies used the BRIEF assessment to score and report the impact of technology-based interventions on planning and organization. No significant effect was found for planning and organization for parent-rated planning and organization.

Two studies reported the BRIEF scores for organizing materials. No significant result was observed for parent-rated organizing materials.

Two studies reported the BRIEF scores for monitoring. The results of parent-rated monitoring were not statistically significant.

Four studies explored the effects of technology-based interventions on metacognition using BRIEF. The results for parent-rated and teacher-rated metacognition were not statistically significant.

The results of moderator analysis showed no moderating effects among the moderators (Table 4).

#### Disruptive Behavior Disorder

Conduct disorder and oppositional defiant disorder are the most common disruptive behavior disorders. Seven studies reported the effectiveness of technology-based interventions on disruptive behavior disorder. Moderate heterogeneity was observed among these studies (*I*^2^=33.7%). Table 3 shows the pooled results of disruptive behavior disorder. Five different scales were adopted: ADHD-RS, Behavioral Observation of Students in Schools, Child Behavior Checklist, DBDRS, and German Rating Scale for Oppositional Defiant and Conduct Disorders. Small and significant effects were observed for parent-rated disruptive behavior disorder measured by Child Behavior Checklist (*K*=3; SMD −0.50, 95% CI −0.72 to −0.29) and DBDRS (*K*=5; SMD −0.31, 95% CI −0.55 to −0.06). Parent-rated disruptive behavior disorder measured by ADHD-RS (*K*=1), Behavioral Observation of Students in Schools (*K*=2), and German Rating Scale for Oppositional Defiant and Conduct Disorders (*K*=3) had no significant effect. No significant effect was observed for teacher-rated disruptive behavior disorder measured using ADHD-RS and DBDRS.

The results of the moderator analysis demonstrated that the number of sessions, sample size, setting, game elements, and type of control group moderated the effect size (Table 4). The number of sessions of >60 (*K*=2; SMD −0.47, 95% CI −0.72 to −0.22; *P*<.001), sample size of ≤50 (*K*=5; SMD −0.40, 95% CI −0.70 to −0.09; *P*<.01), and >100 (*K*=4; SMD −0.38, 95% CI −0.58 to −0.18; *P*<.001); nonhome setting (*K*=4; SMD −0.36, 95% CI −0.02 to −0.62; *P*<.005); game elements excluded (*K*=19; SMD −0.20, 95% CI −0.34 to −0.06; *P*<.006); and no treatment (*K*=10; SMD −0.24, 95% CI −0.41 to −0.06; *P*<.009) moderated the effect size.

#### Visual Attention

Eight studies explored the effects of technology-based visual attention. There was a small heterogeneity among these studies (*I*^2^=22.9%). Table 3 shows the pooled results of visual attention. Four studies used the Continuous Performance Test as measurement, whereas the other study used the Benton Visual Retention Test, Phonological and Visuospatial Reordering, and Integrated Visual and Auditory Continuous Performance Test (IVA/CPT). In addition, 1 study used the Attention Network Test, Attention Switching Task, Rapid Visual Information Processing, Intraextra Dimensional Set Shift, and Reaction Time to score visual attention. Technology-based intervention had small and significant effects on visual attention measured by Continuous Performance Test (*K*=8; SMD −0.42, 95% CI −0.66 to −0.18) and Reaction Time (*K*=2; SMD −0.43, 95% CI −0.79 to −0.06). No significant effect was observed for visual attention measured using Attention Network Test, Attention Network Test, Benton Visual Retention Test, Intraextra Dimensional Set Shift, IVA/CPT, and Rapid Visual Information Processing.

The results of the moderator analysis showed that the number of sessions, sample size, setting, game elements, and type of control group moderated the effect size (Table 4). The number of sessions of >20 to 40 (*K*=18; SMD −0.23, 95% CI −0.38 to −0.08; *P*<.003), sample size of >than 50 to 100 (*K*=27; SMD −0.19, 95% CI −0.30 to −0.07; *P*<.002), mixed setting (ie, combining both home and school or clinic; *K*=8; SMD −0.32, 95% CI −0.57 to −0.08; *P*<.01), game elements excluded (*K*=27; SMD −0.19, 95% CI −0.30 to −0.07; *P*<.002), and equivalent treatment (*K*=13; SMD −0.27, 95% CI −0.44 to −0.10; *P*<.002) moderated the effect size.

#### Auditory Attention

Two studies evaluated auditory attention, which was scored using 2 scales, namely, Children’s Auditory Verbal Learning Test-2 (CAVLT-2) and IVA/CPT. No statistically significant effect was observed for auditory attention measured using these 2 scales (Table 3). Moderate heterogeneity was observed among these studies (*I^2^*=31.3%).

The results of moderator analysis showed no moderating effects among the moderators (Table 4).

#### Sensitivity to Punishment and Sensitivity to Reward

The study by Dovis et al [19] reported the Sensitivity to Punishment and Sensitivity to Reward Questionnaire for Children score. A nonsignificant effect was found for parent-rated sensitivity to punishment and sensitivity to reward (Table 3). Homogeneity was observed for these records (Table 4).

#### Quality of Life

Dovis et al [19] reported the Pediatric Quality of Life Inventory (PedsQL) score. No significant effect was observed for parent-rated quality of life and self-rated quality of life (Table 3). Substantial heterogeneity was observed among these records (*I*^2^=63.1%).

The results of moderator analysis showed no moderating effects among the moderators (Table 4).

#### Reading Fluency

Egeland et al [21] investigated the effectiveness of a technology-based intervention on reading fluency. Logometrica was used to score reading fluency. The result of reading fluency was not at a statistically significant level (Table 3). Small heterogeneity was observed among these records (*I*^2^=13.5%).

The results of moderator analysis showed no moderating effects among the moderators (Table 4).

#### Adherence to Stimulants

Fried et al [44] evaluated adherence to stimulants. There was no significant effect on adherence to stimulants (Table 3). Heterogeneity estimation was not conducted because of 1 effect size in adherence to stimulants. No moderator analysis was conducted because of only 1 effect size for adherence to stimulants (Table 4).

## Discussion

### Principal Findings

The adoption of technologies in school-age children with ADHD has become prevalent in this decade and has been applied in many countries. Nevertheless, the efficacy of applying technology-based treatments for children has not been well established. To the best of our knowledge, this work is the first systematic review and meta-analysis of RCTs to assess the efficacy of technology-based interventions for school-age children, which provides strong evidence for health care practitioners. The pooled results indicated that technology-based treatment has the potential to regulate the inattention, overall executive function, and visual attention of school-age children with ADHD.

### Quality of Evidence

Most of the included studies were of moderate quality, and only 2 studies [25,29] fulfilled the 7 criteria for randomized trials. More than half of the included studies had an unblinded or unclear method of randomization and allocation concealment. Although the blinding of participants was unpreventably broken owing to the nature of the intervention in most studies, the level of influence on the outcomes was considered low in most studies. Only 6 studies were assessed by blinded assessors. The attrition rate in all interventions did not exceed 20%, which met the criteria of the dropout rate. Only 8 studies reported the intention-to-treat analysis. Thus, more rigorous methods should be designed to scientifically justify the effects of technology-based interventions for children with ADHD.

### Effects of Technology-Based Intervention on ADHD Behaviors

The pooled results indicated that technology-based treatment may have the potential to improve computer-rated inattention. No significant results were found for parent-rated and teacher-rated inattention and parent-rated and teacher-rated hyperactivity or impulsivity. This finding is consistent with that of previous studies, which showed that computer-assisted training had a positive effect on inattention [45,46]. Moreover, neurofeedback training and brain-computer interfaces training involve feeding brain activity into a computer, which maps the areas of the brain [47]. The patient controls a computer or video game by producing a brief, sustained brainwave activity in the target area, thereby training the brain regions that are aroused. These treatments include cognitive training to remediate the inattention behaviors of patients with ADHD. The insignificant finding on hyperactivity or impulsivity may be explained by the harmful effect of screen time and the frequent digital media use, which seem to increase the risk of the symptoms of hyperactivity or impulsivity. A meta-analysis conducted by Nikkelen et al [17] identified a modest association between the use of traditional digital media and ADHD behaviors. Notifications and invitation messages may pop up in these training programs. Exposure to these notifications and messages may take away the attention of a child from the task, possibly disrupting the normative development of patience and impulse control [48]. The precise descriptions of computer training, neurofeedback training, and virtual reality have not been completely demonstrated. Thus, relevant information is needed to provide further studies to assess the impact of the messages that suddenly pop up regarding the symptoms of hyperactivity and impulsivity among children with ADHD. Furthermore, it was found that a smaller sample size, nonhome setting, game elements excluded, and no treatment and nonequivalent treatment as the control group tended to have more improving effects on ADHD behavior while conducting the technology-based intervention. This suggests that fewer participants, conducting the intervention at clinic and school, intervention without game elements, and using no treatment or nontechnological intervention as control groups may increase the potential of the technology-based intervention to alleviate ADHD behaviors.

### Effects of Technology-Based Intervention on Executive Functions

Nine categories, namely, inhibition, working memory, flexibility, emotional control, initiation, planning and organization, organizing materials, monitoring, and metacognition, were used to evaluate the executive functions. The pooled result showed a statistically significant effect of the technology-based intervention on parent-rated executive functions. A large significant effect of the control group on computer-rated working memory compared with the technological intervention was found, indicating computed-rated working memory would favor the control group instead of technological intervention. No significant effects were found from other categories related to executive functions. These findings were unexpected, as most previous studies reported positive effects of technology-based training on the improvement of executive functions [18,49,50].

### Effects of Technology-Based Intervention on Disruptive Behavior Disorder

Evidence indicated that technology-based interventions and computer-assisted training may regulate both parent-rated disruptive behavior disorders. This finding is consistent with the previous case study by Kotwal et al [51], who found that the frequency of parent-reported disruptive behaviors in a boy with ADHD was reduced at home and school after conducting a 3-month computer-assisted training. More number of sessions, small and large sample sizes, nonhome settings, game elements excluded, and no treatment as the control group seemed to improve the disruptive behavior disorder when adopting the technology-based intervention. It is unexpected that small and large sample sizes might moderate the association between technology-based interventions and disruptive behavior disorder. Thus, further investigations need to be conducted to evaluate the moderating effect of different sample sizes.

### Effects of Technology-Based Intervention on Visual Attention

Participants’ ability to take in important visual information seems to be significantly improved after conducting computer-assisted training. Ordikhani-Seyedlar et al [52] used brain-computer interfaces with electroencephalograms to generate neurofeedback that significantly improved the visual attention of patients with ADHD. In addition, numerous studies have reported the positive outcomes of adopting neurofeedback training in patients with ADHD [53,54]. Alpha-beta activities in the electroencephalogram have been used to evaluate the attention level of the participants [52]. Furthermore, moderate sessions, moderate sample size, mixed setting, and game elements excluded and using equivalent treatment as a control might improve visual attention under the technology-based interventions. It is somewhat surprising that a mixed setting and equivalent treatment as the control group may have a moderating effect; thus, more RCTs should be conducted to identify the underlying reasons.

### Effects of Technology-Based Intervention on Auditory Attention

The pooled result showed a nonsignificant result on auditory attention. However, some experimental studies have reported the significant effect of biofeedback technology on strengthening auditory attention in children with ADHD [53]. Auditory attention among children with ADHD may be influenced by the intensity and frequency of sound [54,55]. White noise therapy has the potential to improve speech recognition and auditory attention in children with ADHD [56,57]. White noise, which is a random signal with equal intensity at different frequencies, is a steady, unchanging, and unobtrusive sound from certain machines such as a whirring fan and a static radio. The intensity and frequency of sound can remarkably influence the auditory attention of children with ADHD.

### Effects of Technology-Based Intervention on Reading Fluency

No statistically significant effect was found for reading fluency. Nevertheless, previous experiments reported an improvement in oral reading fluency through a computerized program, Headsprout [58]. The computerized program could enhance the engagement of students with ADHD. Hence, learning among these students becomes more effective. This observation also implies that technology, such as a computerized program, has a high potential to enhance oral reading fluency. More RCTs with a larger pediatric population are needed to evaluate the effects of technology on reading fluency among children with ADHD.

### Effects of Technology-Based Intervention on Adherence to Stimulants

Our result regarding the effects of the technology-based intervention on adherence to stimulants was not statistically significant. The literature review conducted by Chacko et al [59] indicated that technology-based interventions could reduce environmental barriers, such as caregivers’ forgetfulness, to improve medication adherence for the pediatric population with ADHD. This nonsignificant finding may be explained by the fact that only a small number of RCTs were conducted to determine the effects of using technologies on stimulant adherence in children with ADHD.

### Effects of Technology-Based Intervention on Sensitivity to Punishment, Sensitivity to Reward, and Quality of Life

A narrative review and pooled results found that technology had no significant effects on sensitivity to punishment and sensitivity to reward and quality of life. Few qualitative and quantitative studies have been conducted to investigate the effectiveness of technology-based interventions on sensitivity to punishment and sensitivity to reward and quality of life among children with ADHD. This circumstance indicates that more trials should be conducted.

### Limitations and Future Research

This study is the first systematic review and meta-analysis of RCTs to examine the effectiveness of technology-based treatment for school-age children with ADHD. The strengths of this review include the use of well-defined inclusive and exclusive criteria, the application of a rigorous and well-constructed search strategy from the 7 electronic databases, and the stringent quality assessment of selected RCTs. However, this study has some limitations. First, the small sample size of the included trials implies the sparsity of RCTs in the children-computer interaction research field. Second, the pooled results may be influenced by variations in intervention designs (ie, intervention intervals and duration) because of the considerable clinical heterogeneity. Third, more than half of the included RCTs were designed using a nonblind approach owing to the unavoidable physical component of the interventions. To minimize these biases, blinded outcome assessors are strongly recommended for open-label RCTs. Fourth, the age selection was between 6 and 12 years, which may exclude preschool age children and adolescents with ADHD benefiting from technology-based treatment. Finally, RCTs published in languages other than English were excluded. Thus, language bias may be premeditated. Although RCT provide excellent internal validity and the most reliable evidence, the results are needed to be interpreted with caution owing to the inclusion of the designated population in this review. Furthermore, the use of virtual reality–based treatments for children with ADHD has been gradually implemented in the field of developmental psychology. RCTs assessing the effects of virtual reality–based treatments on children are still limited; thus, more relevant research is recommended. Moreover, the safety issues of children’s interactions with technological interventions have rarely been discussed in RCTs. Measurements of side effects can be adopted in studies to further optimize the designs of the technological treatment, which can facilitate the universality of technological therapies in patients of different ages.

### Conclusions

This review synthesized evidence from 19 RCTs on the application of technology-based interventions in school-age children with ADHD. The results indicated that the existing RCTs were mainly of low to moderate quality, particularly in random sequence generation, allocation of concealment and blinding, and intention-to-treat analysis. Further well-designed and rigorous trials should be conducted to determine the effectiveness of technology-based interventions in children with ADHD. The pooled results indicated that children’s intervention with technological treatments has effects on computer-rated inattention, parent-rated overall executive functions, parent-rated disruptive behavior disorder, and visual attention. The number of sample sizes, setting of the intervention, exclusion of game elements, and type of control group had moderating effects on ADHD behaviors, disruptive behavior disorder, and visual attention. The potential of technology-based therapies for hyperactivity or impulsivity, inhibition, working memory, flexibility, emotional control, initiation, planning and organization, organizing materials, monitoring, metacognition, disruptive behavior disorder, visual attention, auditory attention, sensitivity to punishment and reward, quality of life, adherence to stimulants, and reading fluency requires further evaluation.

## Appendix

Multimedia Appendix 1 Full search strategy.

Multimedia Appendix 2 PRISMA (Preferred Reporting Items for Systematic Reviews and Meta-Analyses) 2020 checklist.

## Abbreviations

ADHD: attention-deficit/hyperactivity disorder
ADHD-RS: ADHD Rating Scale
BRIEF: Behavior Rating Inventory of Executive Function
CAVLT-2: Children’s Auditory Verbal Learning Test-2
Conners 3-P: Conners 3 Parent Rating Scale
Conners 3-T: Conners 3 Teacher Rating Scale
DBDRS: Disruptive Behavior Disorder Rating Scale
IVA/CPT: Integrated Visual and Auditory Continuous Performance Test
PedsQL: Pediatric Quality of Life Inventory
PRISMA: Preferred Reporting Items for Systematic Reviews and Meta-Analyses
RCT: randomized controlled trial
SMD: standardized mean difference
WISC-IV: Backward Digit Span of the Wechsler Intelligence Scale for Children-IV
