# Supplementary material for: Effectiveness of Technology-Based Interventions for School-Age Children With Attention-Deficit/Hyperactivity Disorder: Systematic Review and Meta-Analysis of Randomized Controlled Trials
Source: JMIR Ment Health. 2023 Nov 21;10:e51459. doi: 10.2196/51459 (PMC10698651; doi:10.2196/51459)
Supplement: Multimedia Appendix 1 [file mental_v10i1e51459_app1.docx]

**PubMed**

#1 ((ADHD) OR (children with ADHD) OR (school-age children with ADHD) OR (students with ADHD)) AND ((technology) OR (computer) OR (robots) OR (virtual reality) OR (VR) OR (augmented reality) OR (AR) OR (web-based) OR (web) OR (serious games)) AND ((inattentive) OR (hyperactive-impulsive) OR (hyperactivity) OR (impulsivity) OR (executive functions) OR (executive functioning) OR (inhibition) OR (working memory) OR (emotional control) OR (flexibility) OR (attention) OR (initiation) OR (planning) OR (organisation) OR (organization) OR (time management) OR (metacognition) OR (quality of life) OR (performance))

#2 ((ADHD[MeSH Terms]) OR (children with ADHD[MeSH Terms]) OR (school-age children with ADHD[MeSH Terms]) OR (students with ADHD[MeSH Terms])) AND ((technology[MeSH Terms]) OR (computer[MeSH Terms]) OR (robots[MeSH Terms]) OR (virtual reality[MeSH Terms]) OR (VR[MeSH Terms]) OR (augmented reality[MeSH Terms]) OR (AR[MeSH Terms]) OR (web-based[MeSH Terms]) OR (web[MeSH Terms]) OR (serious games[MeSH Terms])) AND ((inattentive[MeSH Terms]) OR (hyperactive-impulsive[MeSH Terms]) OR (hyperactivity[MeSH Terms]) OR (impulsivity[MeSH Terms]) OR (executive functions[MeSH Terms]) OR (executive functioning[MeSH Terms]) OR (inhibition[MeSH Terms]) OR (working memory[MeSH Terms]) OR (emotional control[MeSH Terms]) OR (flexibility[MeSH Terms]) OR (attention[MeSH Terms]) OR (initiation[MeSH Terms]) OR (planning[MeSH Terms]) OR (organisation[MeSH Terms]) OR (organization[MeSH Terms]) OR (time management[MeSH Terms]) OR (metacognitio[MeSH Terms]n) OR (quality of life[MeSH Terms]) OR (performance[MeSH Terms]))

#3 ((ADHD[Title/Abstract]) OR (children with ADHD[Title/Abstract]) OR (school-age children with ADHD[Title/Abstract]) OR (students with ADHD[Title/Abstract])) AND ((technology[Title/Abstract]) OR (computer[Title/Abstract]) OR (robots[Title/Abstract]) OR (virtual reality[Title/Abstract]) OR (VR[Title/Abstract]) OR (augmented reality[Title/Abstract]) OR (AR[Title/Abstract]) OR (web-based[Title/Abstract]) OR (web[Title/Abstract]) OR (serious games[Title/Abstract])) AND ((inattentive[Title/Abstract]) OR (hyperactive-impulsive[Title/Abstract]) OR (hyperactivity[Title/Abstract]) OR (impulsivity[Title/Abstract]) OR (executive functions[Title/Abstract]) OR (executive functioning[Title/Abstract]) OR (inhibition[Title/Abstract]) OR (working memory[Title/Abstract]) OR (emotional control[Title/Abstract]) OR (flexibility[Title/Abstract]) OR (attention[Title/Abstract]) OR (initiation[Title/Abstract]) OR (planning[Title/Abstract]) OR (organisation[Title/Abstract]) OR (organization[Title/Abstract]) OR (time management[Title/Abstract]) OR (metacognition[Title/Abstract]) OR (quality of life[Title/Abstract]) OR (performance[Title/Abstract]))

**Search profile PsycInfo**

#1: (TI (“ADHD” OR “children with ADHD” OR “school-age children with ADHD” OR “students with ADHD”))

#2: (TI (“technology” OR “computer” or “robots” OR “virtual reality” OR “VR” OR “augmented reality” OR “AR” OR “web-based” OR “serious games”))

#3: (TI (“inattentive” OR “hyperactive-impulsive” OR “hyperactivity” OR “impulsivity” OR “executive functions” OR “executive functioning” OR “inhibition” OR “working memory” OR “emotional control” or “flexibility” OR “attention” OR “initiation” OR “planning” OR “organization” OR “time management” OR “metacognition” OR “quality of life” OR “performance”))

#4: ((TI (“ADHD” OR “children with ADHD” OR “school-age children with ADHD” OR “students with ADHD”)) AND (TI (“technology” OR “computer” or “robots” OR “virtual reality” OR “VR” OR “augmented reality” OR “AR” OR “web-based” OR “serious games”)) AND (TI (“inattentive” OR “hyperactive-impulsive” OR “hyperactivity” OR “impulsivity” OR “executive functions” OR “executive functioning” OR “inhibition” OR “working memory” OR “emotional control” or “flexibility” OR “attention” OR “initiation” OR “planning” OR “organization” OR “time management” OR “metacognition” OR “quality of life” OR “performance”)))

**EMBASE & Cochrane Library**

(‘randomized controlled trial’/exp OR ‘clinical trial’/exp OR ‘comparative study’/exp OR random*:ab,ti OR control*:ab,ti OR ‘intervention study’:ab,ti OR ‘experimental study’:ab,ti OR ‘comparative study’:ab,ti OR trial:ab,ti OR evaluat*:ab,ti OR ‘before and after’:ab,ti OR ‘interrupted time series’:ab,ti) NOT (‘animal’/exp NOT ‘human’/exp)

#1: (‘ADHD’/exp OR ‘children with ADHD’/exp OR ‘school-age children with ADHD’/exp OR ‘students with ADHD’/exp)

#2: (‘technology’/exp OR ‘computer’/exp OR ‘robots’/exp OR ‘virtual reality’/exp OR ‘VR’/exp OR ‘augmented reality’/exp OR ‘AR’/exp OR ‘web-based’/exp OR ‘serious games’/exp)

#3: (‘inattentive’/exp OR ‘hyperactive-impulsive’/exp OR ‘hyperactivity’/exp OR ‘impulsivity’/exp OR ‘executive functions’/exp OR ‘executive functioning’/exp OR ‘inhibition’/exp OR ‘working memory’/exp OR ‘emotional control’/exp OR ‘flexibility’/exp OR ‘attention’/exp OR ‘initiation’/exp OR ‘planning’/exp OR ‘organization’/exp OR ‘time management’/exp OR ‘metacognition’/exp OR ‘quality of life’/exp OR ‘performance’/exp)

#4: (‘ADHD’/exp OR ‘children with ADHD’/exp OR ‘school-age children with ADHD’/exp OR ‘students with ADHD’/exp) AND (‘technology’/exp OR ‘computer’/exp OR ‘robots’/exp OR ‘virtual reality’/exp OR ‘VR’/exp OR ‘augmented reality’/exp OR ‘AR’/exp OR ‘web-based’/exp OR ‘serious games’/exp) AND (‘inattentive’/exp OR ‘hyperactive-impulsive’/exp OR ‘hyperactivity’/exp OR ‘impulsivity’/exp OR ‘executive functions’/exp OR ‘executive functioning’/exp OR ‘inhibition’/exp OR ‘working memory’/exp OR ‘emotional control’/exp OR ‘flexibility’/exp OR ‘attention’/exp OR ‘initiation’/exp OR ‘planning’/exp OR ‘organization’/exp OR ‘time management’/exp OR ‘metacognition’/exp OR ‘quality of life’/exp OR ‘performance’/exp)

#5: (‘ADHD’:ab,ti OR ‘children with ADHD’:ab,ti OR ‘school-age children with ADHD’:ab,ti OR ‘students with ADHD’:ab,ti) AND (‘technology’:ab,ti OR ‘computer’:ab,ti OR ‘robots’:ab,ti OR ‘virtual reality’:ab,ti OR ‘VR’:ab,ti OR ‘augmented reality’:ab,ti OR ‘AR’:ab,ti OR ‘web-based’:ab,ti OR ‘serious games’:ab,ti) AND (‘inattentive’:ab,ti OR ‘hyperactive-impulsive’:ab,ti OR ‘hyperactivity’:ab,ti OR ‘impulsivity’:ab,ti OR ‘executive functions’:ab,ti OR ‘executive functioning’:ab,tiOR ‘inhibition’:ab,ti OR ‘working memory’:ab,ti OR ‘emotional control’:ab,ti OR ‘flexibility’:ab,ti OR ‘attention’:ab,ti OR ‘initiation’:ab,ti OR ‘planning’:ab,ti OR ‘organization’:ab,ti OR ‘time management’:ab,ti OR ‘metacognition’:ab,ti OR ‘quality of life’:ab,ti OR ‘performance’:ab,ti)
